# Cushing’s syndrome during pregnancy - two case reports

**DOI:** 10.3389/fendo.2024.1326496

**Published:** 2024-03-12

**Authors:** Sumita Cholekho, Yuke Liu, Huiwen Tan

**Affiliations:** Division of Endocrinology and Metabolism, West China Hospital, Sichuan University, Chengdu, China

**Keywords:** Cushing syndrome, pregnancy, hypothalamic- pituitary-adrenal axis, maternal fetal complications, adrenocorticotropic hormone

## Abstract

**Background:**

Cushing’s syndrome (CS) during pregnancy is a rare endocrine disorder characterized by hypercortisolism, which is significantly associated with maternal-fetal complications. Despite its rarity, CS during pregnancy may be related to a high risk of complications for both the mother and fetus.The aim of the present case study is to update the diagnostic approach to CS during pregnancy and the therapeutic strategies for this medical condition to minimize maternal-fetal complications.

**Methods:**

Here, we present two cases of CS in pregnant women, one of whom had twins. Typical clinical symptoms and signs of hypercortisolism developed at the beginning of pregnancy. The plasma cortisol diurnal rhythm of the pregnant patient was absent. CS was confirmed by cortisol and adrenocorticotropic hormone (ACTH) assessment, as well as imaging examination. We investigated the changes in the hypothalamic-pituitary-adrenal axis during normal pregnancy and the etiology, diagnosis and treatment of CS during pregnancy.

**Conclusion:**

Due to the associated risks of laparoscopic adrenalectomy,it is uncertain whether this treatment significantly decreases overall maternal mortality. Additional observational research and validation through randomized controlled trials (RCTs) are required. We advise that CS in pregnant women be diagnosed and treated by experienced teams in relevant departments and medical centers.

## 1 Introduction

Cushing’s syndrome (CS) is an endocrine disorder characterized by hypercortisolism with various causes. CS during pregnancy is rare but troublesome. There are fewer than 200 reported cases of this syndrome during pregnancy in the literature (1). Gonadotropin synthesis could be inhibited by excessive glucocorticoid secretion in female patients with CS during pregnancy, resulting in disorders in ovarian and endometrial functions. They are prone to oligomenorrhea, irregular menstruation and amenorrhea, and trouble getting pregnany. Additionally, expectant individuals with Cushing’s syndrome face a notably elevated risk of experiencing severe complications throughout pregnancy. Long-term exposure to hypercortisolism may cause maternal hypertension, hypokalemia, centripetal obesity, abnormal glucose metabolism, heart failure, pulmonary edema, opportunistic infections, pathological osteoporosis, bone fracture, and even death.The primary cause of CS in pregnant women is adrenal adenoma, followed by Cushing’s disease and ectopic adrenocorticotropic hormone (ACTH) syndrome (EAS). Adrenal adenoma occurs in 15% of nonpregnant women with CS and approximately 50% of pregnant women (2–4). The diagnosis of CS during pregnancy is challenging. Misdiagnosis of CS is also common because it can easily be confused with preeclampsia or gestational diabetes mellitus (GDM). CS during pregnancy may result in maternal-fetal complications. Not only its manifestations, including hypertension and hyperglycemia but also the underlying causes of CS require effective treatment in pregnant women.

Here, we present two cases of Cushing’s syndrome (CS) during pregnancy. One of the pregnant women with CS was expecting twins. The other pregnant woman was diagnosed with ACTH-independent CS and underwent a successful unilateral laparoscopic adrenalectomy. The objective of this study is to describe therapeutic strategies for managing CS during pregnancy to minimize both maternal and fetal complications.

## 2 Case report

### 2.1 Case one

**Figure 1:**
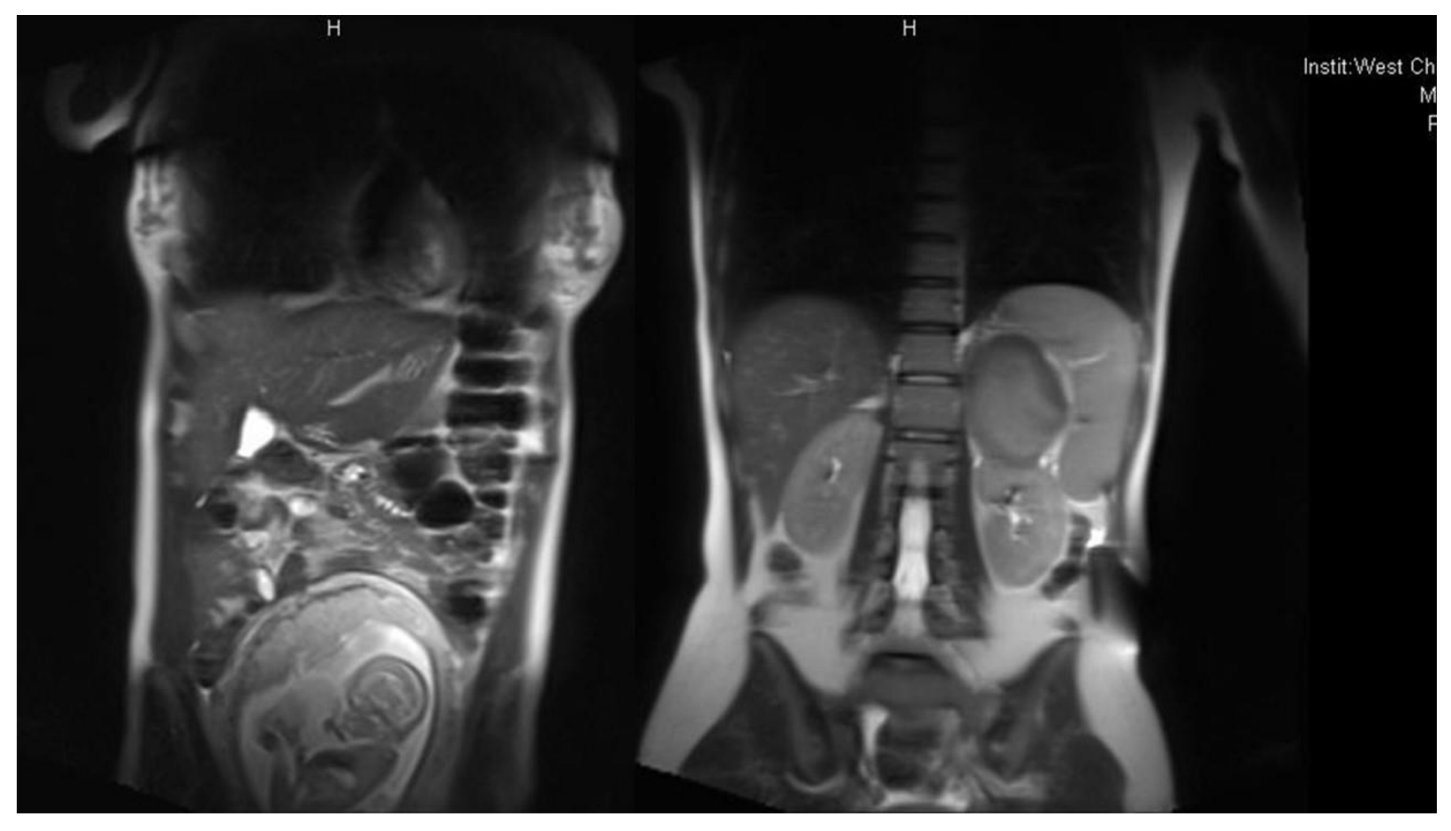
MRI of the adrenal adenoma in a woman with CS during pregnancy.

A28-year-old woman presented with abdominal striae and abnormal glycemic metabolism at 31 weeks of pregnancy. She was admitted to the West China Hospital of Sichuan University for gestational diabetes mellitus (GDM) and suspected Cushing’s syndrome (CS) during pregnancy. During the physical examination, her blood pressure was measured at 132/80 mmHg. She exhibited physical characteristics such as a moon face, buffalo hump, central obesity with a body mass index (BMI) of 28.9 kg/m², abdominal purple striations over 1 cm wide, and mild lower extremity edema. Given these signs, CS was suspected, leading to a referral for laboratory and functional tests related to CS. Hormonal analyses revealed serum cortisol concentrations of 892.26 nmol/L, 759.01 nmol/L, and 929.39 nmol/L at 8:00, 16:00, and 24:00, respectively (normal reference range: 118 ~ 618 nmol/L). Morning serum ACTH was <0.1 ng/L (normal range: 5 ~ 78 ng/L). Midnight saliva cortisol concentrations on three different dates were 66.89 nmol/L, 43.12 nmol/L, and 59.95 nmol/L (normal reference range: 0-10.4 nmol/L). Additionally, 24-hour urinary free cortisol (UFC) concentrations were 723.1 ug/24h and 591.2 ug/24h on two separate occasions (reference range: 20.26-127.55 ug/24h) (see **Table 1**). A 2 mg low-dose dexamethasone suppression test (LDDST) resulted in a serum cortisol concentration of 68.5 nmol/L, and an 8 mg high-dose dexamethasone suppression test (HDDST) showed a concentration of 48.7 nmol/L.Her liver and renal functions and electrolytes were normal. The patient was diagnosed with ACTH-independent CS, and the primary diagnosis was confirmed by magnetic resonance imaging (MRI), which showed a 2.6×2.5 cm adenoma in her leftadrenal gland (**Figure 1**). There were no abnormalities on fetal ultrasound imaging.

**Table 1:** Laboratory findings and HPA of the pregnant patient with CS on admission.

Component	Case 1	Case 2	Reference value and range
*Blood cells count*			
Red blood cells (× 10^12^/L)	3.97	4.36	3.5-5.5
White blood cells (× 10^9^/L)	5.95	7.92	4-10
Platelets (× 10^9^/L)	216	327	100-300
Hemoglobin (g/L)	112	123	120-160
*Glucose metabolism and Lipids*			
Triglyceride(mmol/L)	1.95	2.36	0.29-1.83
Total cholesterol (mmol/L)	4.78	5.65	2.8-5.7
Fasting Plasma Glucose (mmol/L)	4.9	5.18	3.9-5.6
2h Blood glucose (mmol/L)	–	11.94	3.9 -7.8
HbA1c (%)	–	6.6	4.5-6.1
*hypothalamic-pituitary-adrenal axis*			
ACTH (ng/L)	<0.1	1.15	5-78
cortisol 8:00 (nmol/L)	892. 26	345.55	118 ~ 618
cortisol 16:00 (nmol/L)	759. 01	426.97	118 ~ 618
cortisol 24:00 (nmol/L)	929. 39	450.43	118 ~ 618
*Urinalysis*			
Protein	(-)	(-)	negative
Blood	(-)	(+/-)	negative
Glucose	(-)	(+)	negative
24h UFC (ug/24h)	723.1	585.9	20.26-127.55
24h UFC (ug/24h)	591.2	738.6	20.26-127.55

The female patient successfully underwent a unilateral laparoscopic adrenalectomy at 32 weeks of gestation. Adrenalectomy was performed under general anesthesia; during the operation, she was placed in the right lateral decubitus position. No complications developed during surgery. The pathological examination of the removed adenoma revealed adrenal cortical adenoma. This was consistent with the diagnosis of ACTH-independent CS. Three days after surgery, her morning serum cortisol concentration at 8:00 was 185 nmol/L, and she also had fatigue and anorexia. She was administered 30 mg of hydrocortisone (20 mg at 8:00 a.m. and 10 mg at 4:00 p.m.) supplementation due to relative adrenal insufficiency. She was discharged 10 days after laparoscopic adrenalectomy. Her serum cortisol concentration was 200.6 nmol/L, and her cortisol level was 46.5nmol/L after a 1mg dexamethasone suppression test (DST). Her replacement therapy was therefore stopped. A follow-up visit performed 8 weeks after discharge showed normal results. Subsequently, she delivered a healthy baby at 39 weeks gestation through normal delivery, with a birth weight of 3.6 kg. Nine months post-delivery, a 1mg DST indicated cortisol concentrations of 105 nmol/L and ACTH 1.40 ng/L (normal range: 5-78 ng/L), signifying continuous remission.

### 2.2 Case two

A 34-year-old G2P1 female patient visited the West China Hospital of Sichuan University, reporting abnormal blood glucose levels for the past 4 years. She had experienced a weight gain of 10 kg in the last year, along with muscle weakness and elevated blood pressure over the past 4 weeks. Four years prior, she had been diagnosed with gestational diabetes mellitus (GDM) during her first pregnancy at a local hospital. Postpartum, she maintained near-normoglycemia through dietary changes, exercise, and other lifestyle interventions. One year ago, the patient developed centripetal obesity, and her attempts at weight control through diet and exercise proved ineffective. An oral glucose tolerance test (OGTT) revealed a 1-hour blood glucose concentration of 5.37 mmol/L and a 2-hour blood glucose concentration of 10.76 mmol/L after a 75g glucose load. Subsequently, she was found to be pregnant again and received treatment for GDM.

During pregnancy, the patient gradually suffered from facial and back acne, abdominal purple lines, increased body hair, and decreased hairline. Laboratory findings and HPA on admission was shown in **Table 1**. Due to safety concerns, the pregnant woman declined further MRI examination. It is crucial to conduct diagnostic analyses for Cushing’s syndrome (CS) early in pregnancy. In the absence of sufficient evaluation and examination for CS, the patient underwent a cesarean section at 33 weeks of gestation, delivering two male infants who required intensive care for10 days and at-home feeding after 1 month. During a subsequent visit, a 1mg dexamethasone suppression test (DST) was conducted, revealing non-inhibition of cortisol. Enhanced abdominal computed tomography (CT) displayed a 22 mm occupation of the left adrenal branch. An MRI of the sellar regionrevealed no abnormalities in the pituitary glands.

Two weeks after delivery, her blood pressure measured 160/100 mmHg, and oral antihypertensive medications proved ineffective. Consequently, she was admitted to the Department of Endocrinology and Metabolism at West China Hospital. A physical examination revealed a blood pressure of 164/110 mmHg, along with symptoms such as moon face, decreased hairline, abdominal obesity, evident neck and back acne, and abdominal purple stripes. Her serum potassium level was 2.32 mmol/L. Results from the oral glucose tolerance test (OGTT) showed blood glucose levels of 5.18 mmol/L at 0 hours and 11.94 mmol/L at 2 hours after meals. Aldosterone in the decubitus position was measured at 11.65 ng/dL (normal range: 4.5-17.5 ng/dL). The supine aldosterone/decubitus renin activity ratio (ARR) was 3.34, within the normal. ACTH<1.00 ng/L (normal range: 5-78 ng/L), cortisol circadian was absent. The 24-hour urinary free cortisol (UFC) levels were 585.9 ug/24h and 738.6 ug/24h, respectively (normal range: 20.26-127.55 ug/24h).

Bone mineral density (BMD) examination showed that the average Z values of the femoral neck and total hip were -1.7 and -1.7, respectively, and the average Z value of L1-L4 was -2.8. Adrenal contrast-enhanced CT revealed a 2.7x2.5 cm soft tissue density nodule of the left adrenal gland, indicating the possibility of a left adrenal adenoma.

The patient was subsequently transferred to the Department of Urology and underwent laparoscopic adrenalectomy on May 11, 2016. The operation was conducted successfully, and the postoperative pathological examination indicated a left adrenal cortical adenoma. Two days after the surgery, she was transferred back to the Department of Endocrinology and Metabolism.

To address adrenal insufficiency following the surgery, glucocorticoid supplementation therapy (prednisone 10 mg in the morning and 5 mg in the afternoon) was initiated. One week later, she was discharged from the hospital with a blood pressure reading of 140/94 mmHg. Her blood potassium level was 3.6 mmol/L, and her ACTH was 1.05 ng/L, with cortisol concentrations within the normal range.

After discharge, the hormone dose was adjusted and gradually reduced during outpatient follow-ups. Two months post-surgery, a re-examination revealed an ACTH level of 18.46 ng/L. Two years later, the ACTH level increased to 45.84 ng/L, and the 24-hour urinary free cortisol (UFC) was measured at 100.9 ug/24h. Additionally, an oral glucose tolerance test (OGTT) displayed fasting blood glucose at 4.62 mmol/L, 2-hour blood glucose at 10.70 mmol/L, HbA1c at 4.9%, and blood potassium at 3.67 mmol/L.The twin brothers are currently thriving and well-developed.

## 3 Discussion

Pregnancy brings about several physiological alterations, one of which involves the activation of the maternal hypothalamic-pituitary-adrenal axis. This study explores uncommon situations and results in expectant women dealing with Cushing’s syndrome (CS). CS is a collection of syndromes arising from prolonged, excessive cortisol secretion or exogenous intake, influenced by various causes.

The initial documentation of Cushing’s syndrome (CS) during pregnancy was presented in 1953 by Hunt and his colleagues (5). In the initial case series, the fetal mortality rate was noted to be 43%. Recent reports have consistently affirmed that complications affecting both the mother and the fetus are prevalent in Cushing’s syndrome (CS) during pregnancy (6). As pregnancy progresses and the pituitary glands undergo growth, there is a natural physiological increase in pituitary-adrenal axis activity with advancing gestational weeks. This results in elevated levels of ACTH, hepatic corticosteroid binding globulin (CBG), increased serum, salivary, and urinary free cortisol, as well as a lack of suppression of cortisol concentration after dexamethasone administration and placental production of ACTH (7). Hence, the elevated cortisol levels induced by pregnancy pose a challenge in diagnosing Cushing’s syndrome (CS). It is frequently misdiagnosed during pregnancy due to its resemblance to gestational diabetes mellitus (GDM).

In our current study, the presence of a typical Cushing’s phenotype, elevated cortisol levels coupled with low ACTH concentrations, and the identification of adrenal adenoma images on abdominal CT collectively resulted in the diagnosis of ACTH-independent Cushing’s syndrome. This case was determined to be Cushing’s syndrome caused by adrenal cortical adenoma, the most prevalent cause of CS in pregnancy. The mechanism behind pregnancy-induced Cushing’s syndrome may involve luteinizing hormone (LH) and human chorionic gonadotropin (HCG) in pregnant women inducing the overexpression of relevant receptors (such as the chorionic gonadotropin receptor), which exerts a similar effect on the ACTH receptor, leading to adrenal hyperplasia and excessive cortisol production (8).

Our patients exhibited typical symptoms and signs of Cushing’s syndrome (CS). The typical clinical manifestations of pregnancy complicated with CS resemble those in nonpregnant patients, including Cushing’s appearance such as moon face and buffalo back, weight gain centripetal obesity, water and sodium retention, and so forth. CS has repercussions for both the mother and the fetus.In fetuses, a recent review indicated a noteworthy rise in fetal mortality and complications among individuals with Cushing’s syndrome (CS). These complications encompass stillbirth, premature birth, spontaneous abortion, neonatal death, fetal intrauterine growth restriction, fetal malformation, and adrenal insufficiency (1). The placenta utilizes 11β-hydroxysteroid dehydrogenase type 2 (11-β-HSD2) enzymes to enhance the metabolism of cortisol, safeguarding the fetus from excessive cortisol levels (9).

In addition to the typical Cushing’s face, the second patient presented not only abnormal glucose tolerance during pregnancy, hypertension, and osteoporosis but also hypokalemia. A review of the literature revealed that there are limited reported cases of pregnancy complicated by Cushing’s syndrome and persistent hypokalemia in China (10). The occurrence of hypokalemia might also be linked to deficiencies in 11-β-HSD2 enzymes in individuals with Cushing’s syndrome. These deficiencies result in decreased conversion of cortisol to inactive corticosteroids and an augmented binding of cortisol to corticosteroid receptors, consequently elevating potassium excretion (11). In this instance, hypokalemia was a recurring issue before surgery, and the Aldosterone-to-Renin Ratio (ARR) was employed to rule out primary aldosteronism. Blood potassium levels normalized within a week after surgery, suggesting a connection between hypokalemia and Cushing’s syndrome induced by adrenal adenoma. Given the infrequency of pregnancy complicated by Cushing’s syndrome, the existing diagnostic criteria are based on empirical observations.

In this case, ACTH was lower than the lower limit of the reference value, indicating that hypercortisolism may still play a major role in the feedback inhibition of the HPA axis. The 24-hour UFC was more than 3 times higher than normal, and the circadian rhythm disappeared, which was in line with the characteristics of typical CS. Currently, there is no established diagnostic standard for pregnancy complicated by Cushing’s syndrome. Typically, a combination of endocrine laboratory tests and imaging examinations is employed to diagnose suspected cases, aiming to confirm Cushing’s syndrome and provide further clarification on its etiology. Following the International Endocrine Society guidelines for diagnosing Cushing’s syndrome, recommended diagnostic measures for pregnant women suspected of having this syndrome include the measurement of midnight serum or salivary cortisol concentrations (at least twice), 24-hour urinary free cortisol (UFC), and the 2 mg low-dose dexamethasone suppression test (LDDST) (12). Plasma ACTH concentration was used for the differential diagnosis of ACTH-dependent and ACTH-independent CS. The preferred method for screening adrenal cortical adenomas is now renal ultrasound examination. In the case of pregnant individuals with suspected pituitary tumors, the safety of using nonenhanced MRI on the fetus has not been fully established (13). There are reports indicating that individuals with suspected Cushing’s syndrome in the early stages of pregnancy received a definitive diagnosis of Cushing’s disease through the use of high-resolution MRI and bilateral inferior petrosal sinus sampling (BIPSS) (14).

The management of Cushing’s syndrome during pregnancy involves both surgical and medicinal approaches. It is advisable for individuals experiencing pregnancy complicated by Cushing’s syndrome to opt for surgical intervention following the early termination of the pregnancy. Typically, laparoscopic adrenalectomy is the preferred initial treatment for those with cortisol-secreting adrenal adenomas. Medication may be considered when the lesion cannot be identified or surgery is contraindicated. Due to the potential risk of spontaneous abortion in early pregnancy post-surgery and the susceptibility of the fetus to various drugs, medication treatment becomes a consideration. Additionally, anesthesia during surgery could potentially lead to premature birth in late pregnancy. Although there have been successful reports of adrenalectomy, it is noted that the laparoscopic approach is deemed safe (6). For pregnant women diagnosed with Cushing’s syndrome in the final weeks of the third trimester, medical treatment administration and the postponement of surgery until after delivery are often recommended.

As indicated in a recent account, a pregnancy case involving Cushing’s syndrome caused by adrenal cortical adenoma was effectively managed through a combination of metyrapone at 0.5 g three times a day and ketoconazole at 0.4 g twice a day (15). The patient declined surgery and opted for oral medication throughout the pregnancy, demonstrating good drug tolerance and no medication-related complications. Eventually, she successfully delivered a healthy baby boy via cesarean section with an Apgar score of 8. No drug-induced teratogenic effects were observed in the neonate. Following delivery, the adenoma was surgically resected. After a five-year follow-up, the prognosis was favorable. In our observational report, the pregnant patient persisted in preserving the fetus and declined surgical intervention for Cushing’s syndrome until a successful delivery. Two years postoperative, both the mother and baby remained in good health.

## 4 Conclusion

In conclusion, the timely diagnosis and treatment of Cushing’s syndrome (CS) are crucial. Both surgery and medication are viable treatment options for CS during pregnancy. Our findings indicate that pregnancy should not be considered an absolute contraindication for laparoscopic adrenalectomy; rather, this procedure can be regarded as a safe and effective treatment for pregnant women with adrenal CS. However, due to the associated risks of laparoscopic adrenalectomy, it remains unclear whether the treatment significantly reduces overall maternal mortality. Further observational studies and confirmation through randomized controlled trials (RCTs) are necessary. We recommend that CS in pregnant women be diagnosed and treated by experienced teams in relevant departments and medical centers.

## Data availability statement

The original contributions presented in the study are included in the article/supplementary material. Further inquiries can be directed to the corresponding author.

## Ethics statement

The studies involving humans were approved by the Ethics Committee of West China Hospital of Sichuan University (approval number 2022-840). The studies were conducted in accordance with the local legislation and institutional requirements. The patient(s)/participant(s) provided written informed consent to participate in this study. Written informed consent was obtained from the individual(s) for the publication of any potentially identifiable images or data included in this article.

## Author contributions

SC: Data curation, Formal analysis, Investigation, Resources, Visualization, Writing – original draft. YL: Investigation, Writing – original draft. HT: Conceptualization, Funding acquisition, Supervision, Validation, Writing – review & editing.
